# Safety and efficacy of omaveloxolone v/s placebo for the treatment of Friedreich's ataxia in patients aged more than 16 years: a systematic review

**DOI:** 10.1186/s13023-024-03474-6

**Published:** 2024-12-30

**Authors:** Ankita Umrao, Monika Pahuja, Nabendu Sekhar Chatterjee

**Affiliations:** Discovery Research Division, Indian Council of Medical Research (ICMR) Headquarters, V. Ramalingaswami Bhawan, Ansari Nagar, P.O. Box 4911, New Delhi, 110029 India

**Keywords:** Omaveloxolone, Friedreich’s ataxia, Rare disease, Systematic review, Randomized clinical trials, mFARS

## Abstract

**Background:**

Friedreich’s ataxia (FA) is a rare genetic disorder caused by silencing of the frataxin gene (*FXN*)*,* which leads to multiorgan damage. Nrf2 is a regulator of *FXN,* which is a modulator of oxidative stress in animals and humans. Omaveloxolone (Omav) is an Nrf2 activator and has been reported to have antioxidative potential in various disease conditions. The present review was conducted to determine the use of Omav, the only FDA-approved treatment for FA.

**Methods:**

Three electronic databases, Cochrane, PubMed and Google Scholar, were searched with terms such as ‘Omaveloxolone’, ‘Friedreich ataxia’, ‘genetic diseases’, ‘autosomal recessive’, and ‘rare disorders’ using various advanced search filters. Articles were screened, extracted, and assessed for quality, and a qualitative synthesis of the data was performed. The study protocol was registered in PROSPERO (CRD42024531449).

**Results:**

A total of 201 records were found, with very few published research articles on the topic. Only two randomized clinical trials published in a series of three research articles were included in the current systematic review. Peak load exercise and modified Friedreich’s Ataxia Rating Scale (mFARS) values were considered the major outcome measures for determining the efficacy of 150 mg Omav capsules/day in FA. Exploratory outcome measures, such as low-contrast letter visual acuity test, exercise test, T25-FW, 9-HPT, health-related quality of life, and biochemical tests, were also assessed along with adverse events in all the studies.

**Conclusion:**

Although, the quality of the articles demonstrated low bias. However, the short duration, small sample size, and missing data, including the values of different measures of mFARS scores in patients, limit the generalizability of the results. Further studies with longer durations and in severe patients with foot deformities are needed to clearly define the efficacy of Omav in FA and to determine the optimal drug for FA patients in India.

**Supplementary Information:**

The online version contains supplementary material available at 10.1186/s13023-024-03474-6.

## Introduction

Friedreich’s ataxia (FA) (OMIM # 229300), a rare degenerative autosomal recessive disorder, is caused by silencing of *FXN* on chromosome 9 by extension of the GAA triplet in its first intron. *FXN* encodes frataxin, a highly conserved mitochondrial protein of 210 amino acids involved in iron-sulfur protein cluster biogenesis [1] and iron homeostasis [2, 3]. A point mutation in FXN leads to the absence of a functional frataxin protein. A decrease in frataxin results in an increase in free radicals and iron, leading to cellular damage, particularly in organs with high energy metabolism, such as the nervous system, cardiac muscles, and pancreas [2]. GAA repeats repress *FXN* transcription. The intensity of the expansion of the triplet determines the severity and progression of the disease [4].

Nrf2 regulates at least 250 genes through antioxidant response elements and shuttles between the nucleus and cytoplasm. Under conditions such as FA, unlike under stress, FA is unable to regulate pathways involved in oxidative stress, inflammation, iron metabolism, lipidosis, and ferroptosis due to its inability to translocate to the nucleus [1, 5]. In the past decade, a variety of antioxidants have been reported to be successful in early studies but failed in phase III studies of FA. The reasons for this failure included a small placebo (PBO) response, the absence of a PBO group, and a short study duration.

The average life expectancy of FA patients is 30–40 years, and the age at onset is 6–18 years [4, 6–9]. To date, there is no cure for this condition, and omaveloxolone (SKYCLARYS) is the first and only U.S. FDA-approved drug (approved in February 2023) available for FA treatment [10]. Omaveloxolone (Omav) is a synthetic oleanane terpenoid nuclear factor erythroid 2-related factor 2 (Nrf2) activator. Omav restores the function of Nrf2 by preventing its ubiquitination, suppressing inflammation and oxidative stress, increasing mitochondrial function, and promoting normal cellular metabolism in FA-derived cells [11].

Omav has been extensively studied in mouse models as an Nrf2 activator in various inflammatory disease conditions, such as ischemic optic neuropathy [12], epidermolysis bullosa [13], sepsis-induced cardiomyopathy [14], intracerebral hemorrhage [15], mitochondrial myopathy [16], osteoarthritis [17], and hepatoprotective activity [18].

Omav protected cerebellar granule neurons collected from a mouse model of FA against hydrogen peroxide-induced oxidative stress and significantly induced Nrf2 by regulating mitochondrial respiration and glutathione concentrations [19, 20]. Pharmacokinetic and pharmacodynamic studies of Omav have also been reported in primates; Omav is distributed in important tissues, including the liver, lung, and brain, and induces glutathione homeostasis, redoxins, and Nqo1, which in turn induces the Nrf2 gene [21].

The National Policy for Rare Diseases, 2021 (NPRD, 2021), has identified three groups of disorders based on clinicians’ expertise, including disorders amenable to one-time curative treatment, disorders requiring long-term/longevity, documented low-cost treatment, and disorders with available definite treatment, but patient selection needs to be optimized for benefit and very high cost. These categories are periodically reviewed by the Central Technical Committee for Rare Diseases (CTCRD) based on scientific updates to provide access to affordable health care for rare disease patients. Considering the availability of treatment for FA in India, the age of FA onset, and the relatively short life expectancy, the inclusion of Friedreich's Ataxia under the NPRD, 2021, is under consideration. Therefore, the Indian Council of Medical Research (ICMR) is commissioned to conduct a systematic review on the use of Omav for the treatment of FA by CTCRD.

## Results

### Data characterization and analyses

**Fig. 1:**
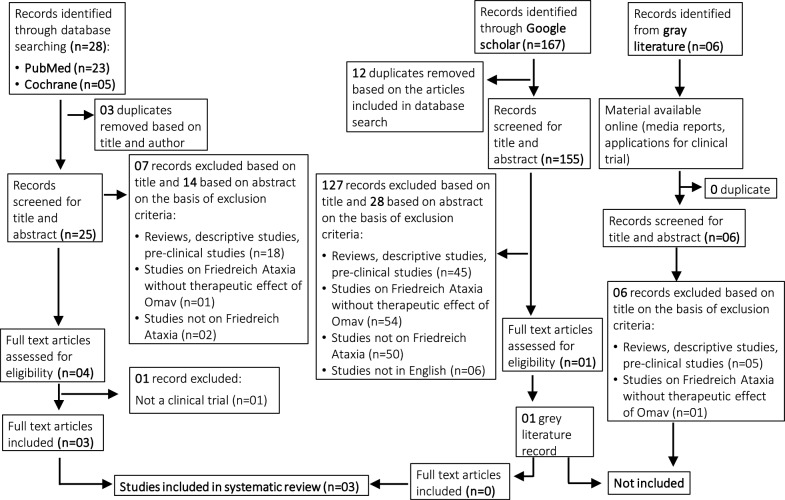
PRISMA flow diagram depicting the search strategy and included and excluded studies in the systematic review

Overall, 201 records were saved from the three electronic databases and the gray literature [see Additional file 2]. After deduplication, 186 records were first screened based on title and abstract (Fig. 1). Furthermore, 182 records were excluded for the following reasons: reviews, descriptive studies, preclinical studies (n = 68), studies on Friedreich’s Ataxia (FA) without the therapeutic effect of omaveloxolone (Omav) (n = 56), studies not on FA (n = 52) and studies not in English (n = 06). In the second screening, five records were assessed based on the full text, out of which two records were excluded based on studies falling in gray literature (n = 01) and not a clinical trial (n = 01). Finally, a total of three articles were included for data extraction. Only clinical trial(s) were included. We found that there were no observational or case studies on the use of Omav for FA patients. Gray literature was excluded. A PRISMA flow chart detailing the sequential stages in screening the records is summarized in Fig. 1. As only one clinical trial (4 articles) was available on patient population, heterogeneity in duration, follow-up, and outcome measures, a meta-analysis could not be performed. Therefore, we provide a qualitative overview of the clinical evidence of the effect of the Omav intervention on FA in the included studies. Part 1 and Part 2 are the only randomized clinical trials (RCTs); therefore, these two studies were included in the quality assessment.

### Risk of bias assessment

Two RCTs, which were published as three peer-reviewed research articles, were part of this systematic review. Part 1 and Part 2 were the only RCTs included in the quality assessment. Both articles had a low risk of bias, with four and two points of certainty, respectively. A summary of the risk of bias and quality assessment is provided in Tables 1 and 2.

**Table 1:** Risk of bias assessment of two included randomized controlled clinical trials

Study	Outcome	Sequence generation	Allocation sequence concealment	Blinding	Missing outcome data	Publication bias
Lynch et al.,2020	Safety	Low	Low	Low	Low	Low
Lynch et al.,2020	Efficacy	Low	Low	Low	Low	Low
Lynch et al.,2022	Safety	Low	Low	Low	Low	Low
Lynch et al.,2022	Efficacy	Low	Low	Low	Low	Low

**Table 2:** Quality assessment of two included randomized controlled clinical trials

No. of studies	Certainty assessment							No. of patients		Effect	Certainty
	Study design	Efficacy outcome	Risk of bias	Inconsistency	Indirectness	Imprecision	Other considerations	Omav^a^	PBO^b^	Relative (95% CI)	
1	Randomized trial	Peak work attained during maximal exercise testing	Low	Low	Low	Low	None	52	17	1.1, 21.9	⊕⊕⊕⊕ High
2	Randomized trial	mFARS^c^ score	Low	Low	Low	Low	None	51	52	− 2.93 to − 0.18	⊕⊕⊕⊕ High

^a^Omaveloxolone treated

^b^Placebo

^c^modified Friedreich’s Ataxia Rating Scale

### Study characteristics

The three included original articles were from one clinical trial that is still ongoing, the MOXIe study (Table 3). The MOXIe study was published in three parts: Part 1 (12 weeks) was a dose-range phase-2, double-blind, randomized, placebo (PBO)-controlled, multicentric trial to assess the pharmacodynamics, safety, and efficacy of Omav in FA; Part 2 (12–52 weeks) was a parallel-group phase-2 interventional, double-blind, randomized, PBO-controlled, multicenter, registrational study to assess the safety and efficacy of Omav in FA; and Part 3 (52–196 weeks or EW144) was an open-label, delayed-start analysis of the MOXIe extension. Parts 1 and 2 are the only RCTs and negligibly vary in study characteristics (Table 3).

**Table 3:** Details of the clinical trial(s) on the use of omaveloxolone for Friedreich’s Ataxia

Clinical trial	Study Design	Study duration/follow-up	Comparator	Sample size	Endpoint	Referencess
MOXIe trial (NCT02255435)	Part 1: Phase 2; double blind; randomized, PBO^a^-controlled, dose-range, international, multicentric study	12 weeks	PBO	N^d^ = 69 PBO = 17 Omav^**b**^ = 52	Safety of various doses of Omav in patients with FA	[10, 22]
	Part 2: Phase 2; double-blind, randomized, PBO controlled, registrational, international, multicentric, parallel-group trial	52 Weeks (48 weeks treatment + 4 weeks follow-up)	PBO	n = 103 PBO = 52 Omav = 51	Safety and efficacy of 150 mg Omav once/day in patients with FA	[22, 23]
	Part 3: Blinded; open-label extension study	196 weeks	Baseline at week 48	n = 82 PBO-Omav = 42 Omav-Omav = 40	Long-term safety and efficacy of Omav patients with FA^c^ following completion of Part 1 or Part 2	[22, 24]

^**a**^Placebo

^**b**^Omaveloxolone treatment

^**c**^Friedreich’s Ataxia

^d^number of patients enrolled

**Fig. 2:**
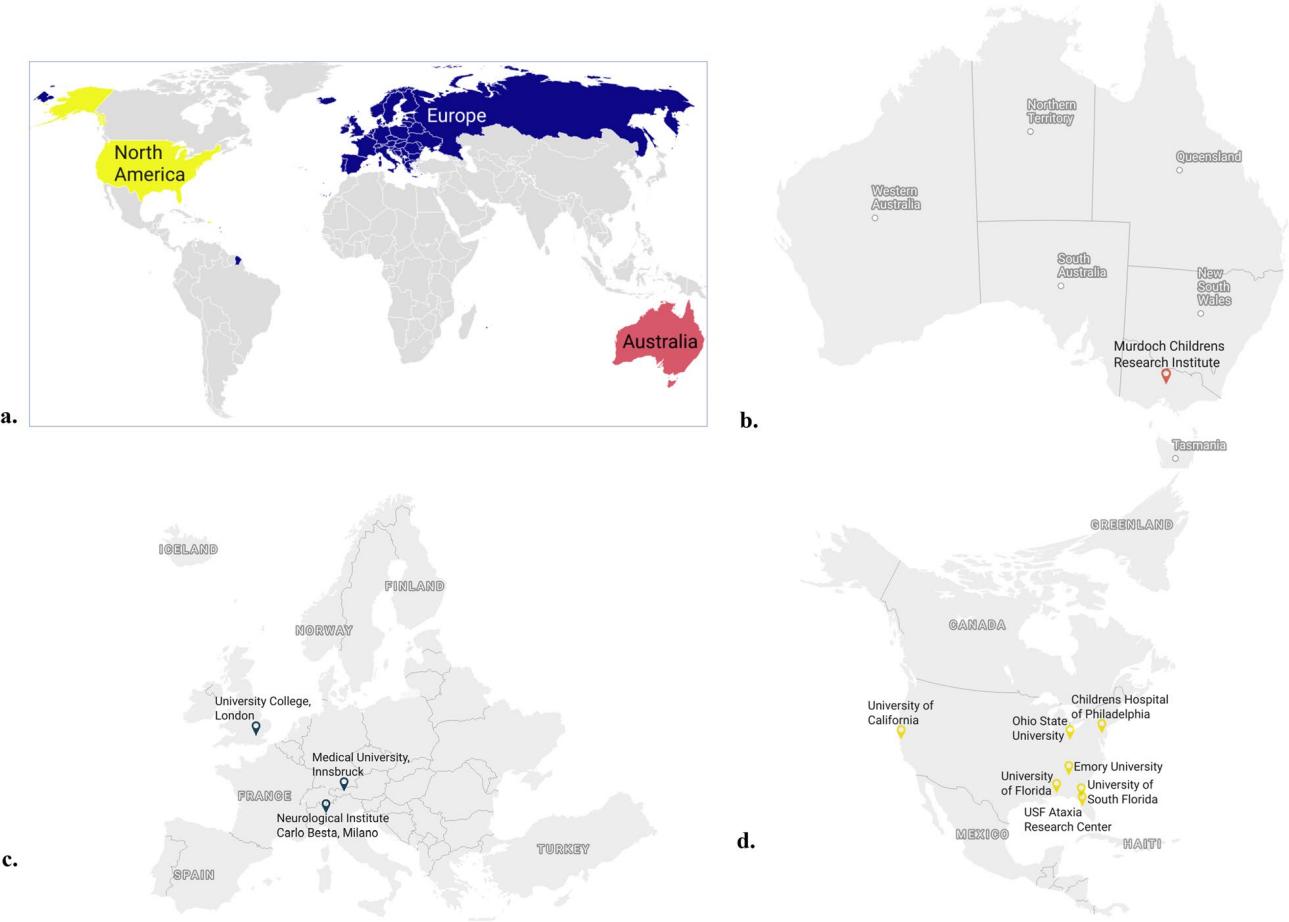
Maps showing the 11 study sites in the clinical trial located on three different continents. **a** Global map showing the three continents from which FA patients were enrolled. Study sites in **b** Australia (1), **c** Europe (3) and **d** North America (7)

The studies were conducted in three continents, namely, Australia (n = 8), Europe [Italy (n = 7), the United Kingdom (n = 8), Austria (n = 9)], and the United States (n = 71), where ‘n’ is the number of patients recruited from each site in the MOXIe trial by March 2022 (Table 3). The locations of the study sites are shown in Fig. 2.

The percentage of overall female participants was 33% in the PBO group and 60–61% in the Omav group. The study population included mostly whites. The patients in both the PBO and Omav groups from part 2 (after week 52) received Omav in part 3, representing PBO-Omav and Omav-Omav, respectively. Overall, 160 mg of Omav once/day was the optimal dose for improving neurological function in Part 1. Therefore, 150 mg once/day Omav treatment was reported for FA efficacy (Table 4). The patients and examiners were blinded during enrollment, throughout the treatment and during follow-up in all the studies.

**Table 4:** Study and treatment characteristics of the studies on the use of omaveloxolone by Friedreich’s Ataxia

Study	Inclusion criteria	Exclusion criteria	Treatment regimen	Randomization
Part 1 [10]	Genetically confirmed FA^a^ with an mFARS^b^ score 10–80 Age: ≥ 16 to ≤ 40 years Ability to complete maximal exercise testing, and able to ride an exercise ergometer (approximately 60 rpm) for 3 min with no additional resistance	History of clinically significant cardiac disease BNP^c^ level > 200 pg/mL Uncontrolled diabetes (HbA1c^d^ > 11.0%)	Cohorts (n = 8) at ascending dose levels for 12 weeks Two cohorts 160 mg once/day) Omav^e^ (n = 12) and PBO^f^ (n = 4), Two cohorts (300 mg once/day) Omav (n = 10) and PBO (n = 3)	3:1::Omav: PBO for each dose
Part 2 [23]	Confirmed disease for 3–5 years; mFARS score 10–80	Same as part 1 and individuals with severe foot deformity	150 mg capsule of Omav once daily	1:1::Omav: PBO Stratified as with pes cavus and without pes cavus
Part 3 [24]	Confirmed disease and completed MOXIe part 2 with follow up mFARS score 20–80	Same as part 2	150 mg capsule of Omav once daily	Not applicable

^a^Friedreich’s ataxia

^b^modified Friedreich’s Ataxia Rating Scale

^c^B-type natriuretic peptide

^d^Hemoglobin A1C test

^e^Omaveloxolone treatment

^f^Placebo

### Patient characteristics

Patients with genetically confirmed FAs were included; however, not all patients had baseline GAA1 repeat length data. The inclusion criteria had negligible variation among all the studies (Table 4). The mean age of the study participants ranged from 16 to 40 years, and the mean disease duration was 3–5 years. The detailed patient characteristics are listed in Table 5.

**Table 5:** Patient characteristics in reported studies

Study	Age at onset (years)	Age at diagnosis (years)	Female	Ethnicity (white)	GAA1 repeat length	Ambulatory status	Pes cavus status	mFARS^a^	History
Part 1 [10]	15.3 ± 4.8 (PBO^b^) 14.8 ± 4.8 (Omav^c^) Mean 25.6	24.4 ± 6.7 (PBO) 25.9 ± 6.4 (Omav)	52% (Omav) 59% (PBO)	94% (PBO) 98% (Omav)	863 ± 278 (PBO) 700 ± 277 (Omav)	94% (PBO) 89% (Omav)	59% (PBO) 42% (Omav)	40.5 ± 10 (PBO) 41.3 ± 12 (Omav)	Areflexia 77% (PBO) 81% (Omav)
									Scoliosis surgery 18% (PBO) 12% (Omav)
Part 2 [23]	15.6 ± 5.3 (PBO) 13.87 ± 4.7 (Omav)	24.1 ± 7.8 (PBO) 23.4 ± 6.1 (Omav)	33% (Omav) 61% (PBO)	96.2% (PBO) 98% (Omav)	676.2 ± 267.9 (PBO) 736.8 ± 206.8 (Omav)	94% (PBO) 90% (Omav)	5.2% (PBO) 5.1% (Omav)	37.8 ± 10.8 (PBO) 40.8 ± 10.2 (Omav)	Cardiomyopathy 29% (PBO) 49% (Omav)
									Scoliosis 71% (PBO) 77% (Omav)
									Scoliosis surgery 19% (PBO) 31% (Omav)
Part 3 [24]	15.1 ± 5.3 (PBO-Omav) 15.9 ± 5.7 (Omav-Omav)	23.6 ± 7.8 (PBO) 24.2 ± 6.5 (Omav)	33% (PBO-Omav) 60% (Omav-Omav)	93% (PBO-Omav) 98% (Omav-Omav)	693.8 ± 277.2 (PBO-Omav) 739.2 ± 214.9 (Omav-Omav)	93% in both the groups	-	38.8 ± 11 (PBO) 40.9 ± 10.4 (Omav)	Cardiomyopathy 29% (PBO-Omav) 48% (Omav-Omav)

Values are mean ± SD

^**a**^mcodified Friedreich’s Ataxia Rating Scale

^b^Placebo

^c^Omaveloxolone treated

All the enrolled patients discontinued all the antioxidant supplements not less than half a month earlier. Patients who later developed cardiac disease (such as arrhythmias) or diabetes remained in the study unless they chose to withdraw.

### Safety and efficacy measures

The primary and secondary outcomes were different in various parts (1–3) of the clinical trial.

#### Primary outcome

In Part 1, the functional neuromuscular assessment (FNM) of Omav was the primary outcome for determining the safety and potential benefit of Omav [10]. FNM was measured by maximal exercise testing, which was assessed as peak oxygen utilization and peak workload, by conducting cycle ergometry using a reclining immobile bicycle. In Part 2 and the extension study, the modified Friedreich’s Ataxia Rating Scale (mFARS) score was considered a functional neurological assessment that included four subsections to measure bulbar function, upright stability, and upper and lower limb coordination. The change from baseline in neurological abilities compared with placebo (PBO) through week 48, extended week (EW) 72, EW 96, EW 120, and EW 144 was used as primary measures to determine the efficacy of Omav [23, 24]. mFARS scores range from 0 to 99; the lower the score is, the better the neurological function.

#### Secondary outcomes

In part 1, the key secondary measure to determine the safety and benefit of Omav was neurological ability [10]. However, in part 2, the efficacy of Omav on FA was the secondary outcome, which was assessed [23, 24] and performed as described earlier [10, 25, 26]. The mFARS score and noninferiority analyses included data from both week 48 and through EW 144.

#### Exploratory measures

Exploratory measures included a few laboratory and biochemical tests to identify the frequency and severity of adverse effects, including a low-contrast letter visual acuity test, exercise test, nine-hole peg test (9-HPT) [3], timed 25-foot walk test (T25-FW) [2] and health-related quality of life [4]. Biochemical parameters, vital signs, physical evaluations, and adverse events at baseline and weeks 1, 2, 4, 8, and 12 were also explored in these studies.

### Clinical recovery

In part 1, a nonsignificant increase in peak workload (the primary outcome measure) was reported at 160 mg once/day by week 12 from baseline [10].

#### mFARS score:

The mFARS scores and noninferiority analysis, including data from both parts 2 and 3, were obtained.

In part 1, Omav improved the mFARS score in a dose- and time-dependent manner, with less improvement at 300 mg/day and a maximum improvement at 160 mg/day. This result was equivalent to the predicted improvement of 1 year in FA, with the maximal response occurring in patients receiving an 80–160 mg/day dose of Omav [10]. In parts 2 and 3, a notable number of mFARS values were missed due to interruptions in on-site clinical visits during the coronavirus disease 2019 (COVID-19) epidemic (Fig. 2) [23, 24]. Apart from part 2 of the mFARS data, close parallel trajectories were observed between both groups. The trajectories in part 3 persisted through EWs 120 and 144. In addition, the annualized slopes in the open-label study through EW 144 for the Omav-Omav [0.45 ± 0.63 (95% CI: 0.82, 1.71)] and PBO-Omav [0.76 ± 0.28 (95% CI: 0.21, 1.31)] groups were not significantly different and demonstrated no convergence. Moreover, the mFARS score in the Omav group was maintained relative to baseline through EW 120 [24].

#### mFARS assessment

**Fig. 3:**
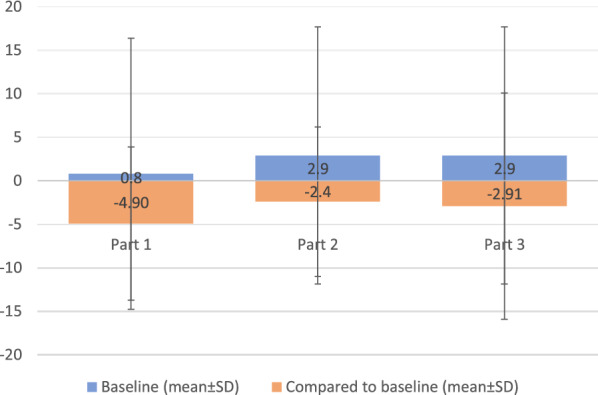
Differences in mFARS values (Omaveloxolone-placebo) (mean ± SD) in part 1 (week 12), part 2 (week 48), and part 3 (EW 72) in patients without pes cavus according to each study. Baseline values in part 1 include the data of patients with pes cavus. The baseline values are the initial values obtained at the time of patient recruitment. The baseline value in part 3 is the same as that in part 2

Omav improved the four subsections involved in the mFARS assessment relative to PBO, with the greatest effect on upright stability. The maximum effect was observed in pediatric patients in part 2, which, when randomized to PBO, worsened and improved when allocated to the Omav group (Fig. 3). The maximum effects were observed in males and at sites in the United States.

In part 1, the absence of pes cavus (FA-associated neuromuscular foot deformity) was associated with greater improvements in mFARS in patients treated with 160 mg/day Omav [4.4 points (*P* = 0.01)]. Compared with those in PBO patients, workload increased significantly by 11.5 W (95% CI 1.1, 21.9) and improved during exercise testing and T25-FW. Other disease features, such as shorter (GAA1) and longer (GAA2) repeat length, disease duration, age, sex, age of onset, prior scoliosis surgery, and ambulation assist type, had no clear response to Omav or correlation with the mFARS score [10].

In part 2, in addition to the case of pes cavus, the inclusion of cardiomyopathy and GAA repeats as covariates improved the effect of Omav on the mFARS score, with a difference of − 3.48 points relative to that of PBO (*p* = 0.0012) between the groups. However, Omav did not significantly improve the mFARS score (Pearson correlation for mFARS versus Patient Global Impression of Change [PGIC]: r = 0.47, *p* < 0.0001; Clinician Global Impression of Change [CGIC]: r = 0.44, *p* < 0.0001) or favor Omav treatment for FA [23].

#### Secondary outcome measures

The mean CGIC and PGIC scores in part 2 did not significantly improve between the groups, and being the first in the hierarchy, the other endpoints did not significantly improve. Similarly, Omav improved activities of daily living (FA-ADL) scores relative to baseline at week 48, with nominal statistical significance relative to PBO [23].

### Adverse events

Safety assessments considered in treating FA with Omav included assessments of patient body weight, basic metabolic indices, laboratory test results (clinical chemistry, hematology, and urinalysis), and physical examinations, including pes cavus, vital sign measurements, associated medications, and adverse effects, including serious adverse events.

**Fig. 4:**
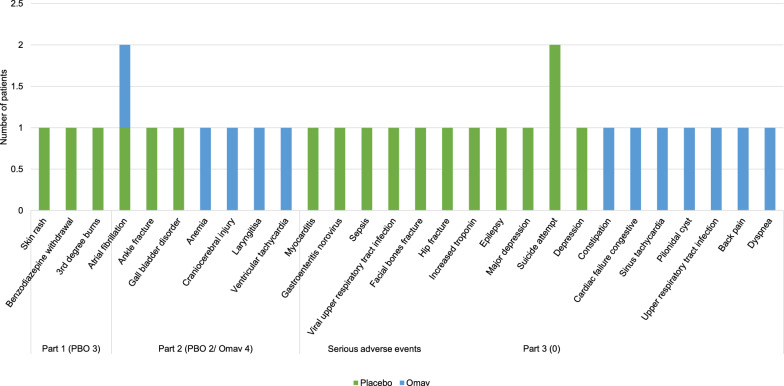
Serious adverse events (SAEs) in part 1 (week 12), part 2 (week 48), and part 3 (EW 196). The numbers on the X-axis are the number of patients who withdrew due to SAEs in the respective studies and groups. Three patients from the placebo group in part 1, two patients from the placebo group, and four from the Omav group in part 2 withdrew from the study. None of the participants withdrew from part 3 due to SAEs

In Part 1, three patients withdrew from the PBO group (Fig. 4) [10]. The incidence of adverse events in Part 2 was similar in all FA patients in both groups with mild to moderate severity [23]. Adverse events were common in the Omav group and included nausea, diarrhea, headache, fatigue, and abdominal pain. Increased nasopharyngitis and upper respiratory tract infections were noted in part 1 only, which was assumed to be due to acclimatization. An increase in aspartate and alanine aminotransferase (AST and ALT, respectively) was persistent throughout the trial; however, the occurrence of other adverse effects continued until the patients adjusted to Omav in part 1 [10].

Adverse events were less frequent in Part 2 and occurred with similar frequency across the Omav and PBO groups (data not available) [23]. AST and ALT returned to baseline levels within four weeks following drug withdrawal in a similar trend. In addition, none of these patients had clinical symptoms or hepatic burdens or met Hy’s law criteria. However, the isolated pharmacological effects of Nrf2 activation are unknown [23].

On average, a mean decrease in weight relative to the PBO or baseline weight was observed in the Omav group at week 48. This effect was limited to patients aged ± 18 years and was distinct in patients with a baseline body mass index > 25 kg/m^2^ (overweight) (data not available) [23].

In part 3, 13 (8.7%) patients experienced SAEs, among whom 5 (11.6%) were in the Omav group and 8 (7.5%) were in the PBO group. Nevertheless, all SAEs were considered unrelated to the study and did not result in the discontinuation of Omav. The majority of patients experienced adverse events related to depression. The most common adverse events caused by Omav treatment were increased coronavirus infection and ALT [24].

In addition, none of the adverse events led to treatment discontinuation in FA patients younger than 18 years of age, and despite the adverse events, no deaths were reported.

### Potential bias

From the start, the laboratory results were only viewed by personnel monitoring adverse events and were blinded to the neurological evaluators to minimize unblinding. However, these results were delivered days after the evaluations were performed.

In addition, hope bias is a complicating factor in investigating mitochondrial diseases. There is a possible presence of such bias in the trial in Part 3, especially in the PBO-Omav group. As the maximal effect in the PBO-Omav group occurred at EW 24, this period was sufficient for reporting the possible symptoms of Omav.

## Methods

This systematic search aimed to summarize the use of omaveloxolone for the treatment of Friedreich’s ataxia (FA). The protocol used to conduct the systematic review was evaluated and registered online by the International Prospective Registry of Systematic Reviews (PROSPERO) with accession number CRD42024531449.

### Literature search

An initial systematic electronic literature search was performed in the Cochrane Library and PubMed/National Library of Medicine (NLM) databases on 26th March 2024 to identify the use of Omav for the treatment of FA from 1st January 2014 to 1st March 2024. The search was also performed through Google Scholar, and gray literature was obtained from open search engines and clinical trial registries (clinicaltrials.gov, the EU Clinical Trials Register and the World Health Organization’s International Clinical Trials Registry Platform [WHO-ICTRP]). However, the data obtained from gray literature were not considered in the systematic review.

### Search strategy

The title was broken down in terms of population, intervention, comparator, outcome(s), and study design (PICOS). As FA is a rare disease and Omav is the only FDA-approved drug to date, carefully chosen words were assigned to explore the population and intervention through controlled vocabularies and a set of operators, as specified in different databases. The search terms used were “MeSH”, “skyclarys”, “children”, “adults”, “young adults”, “Friedreich ataxia” and “Omaveloxolone” [see Additional file 1].

### Screening of studies

After performing the search, all the obtained records were separately downloaded in.csv format in an Excel workbook. Duplicates within and across each downloaded workbook were removed. The resultant records were then screened, assessed, and considered based on the following *inclusion criteria*: (1) studies reporting outcomes for treatment of FA by Omav; (2) full-text available; (3) studies including interventional studies; and (4) studies in English. *The exclusion criteria* were as follows: (1) reviews, descriptive studies, correspondence studies, and preclinical studies; (2) studies on FA without the therapeutic effect of Omav; (3) studies that only consisted of abstracts; and (4) studies that did not involve FA. The screening was performed in another Excel workbook following the Preferred Reporting Items for Systematic Reviews and Meta-Analyses (PRISMA) guidelines. The records were first screened by viewing the title, then reading the abstract, and finally considered by reading the full text for initially screened records. The reason for excluding the records was documented. Similar to the database search, screening was also performed independently by two authors (AU and MP), and any conflicts were resolved by discussion and/or by the intervention of the third author (NC).

### Data extraction

This systematic review was conducted according to the PROSPERO and PRISMA checklists. Only original research articles in the form of clinical trials were included for data extraction. AU extracted the data, and AU and MP independently evaluated the extracted data. The following data were extracted from the studies: study characteristics, patient characteristics, treatment characteristics, primary and secondary outcomes, clinical improvement, disease progression, mortality, mean ± SD values, and 95% confidence intervals (CIs) for efficacy outcomes and safety of Omav. The mean and standard deviation (SD) were calculated from the available data wherever not reported in that format. This review included a narrative summary of the quantitative data and extracted qualitative data whenever reported.

### Quality assessment

To assess the validity of the included records, risk of bias (RoB) and quality assessment were performed by two independent reviewers (AU and MP). The articles published in peer-reviewed journals were grouped as appropriate [randomized clinical trials (RCTs) and experimental studies]. The articles were assessed for the quality of study design, methodology, analysis, and interpretation using the Revised Cochrane risk-of-bias tool (RoB 2) for randomized trials. According to field, the included records were ranked as having high, medium, or low risk of bias.

### Data synthesis

A qualitative summary of the included records was synthesized in this systematic review. The study, patient and treatment characteristics; primary, secondary, and exploratory outcomes; clinical recovery in terms of the mFARS score; adverse events; and potential bias were reported from each included study using descriptive statistics. As the same cohort was included in all the reported studies, the data were not meta-analyzed due to overlapping samples.

## Discussion

Omav is a semisynthetic oleanane triterpenoid and an activator of Nrf2, a master transcription factor for the regulation of anti-inflammatory, antioxidative, and mitochondrial bioenergetic genes. Nrf2 is suppressed in FA patients and therefore suppresses mitochondrial function and diminishes antioxidant levels due to a genetic mutation in frataxin, a protein involved in iron homeostasis. Omav was approved in the United States for medical use in February 2023 and was recommended by the Committee for Medicinal Products for Human Use (CHMP) of the European Medicine Agency for market authorization for the medicinal product Skyclarys, which is intended for treating a rare genetic disorder, FA. In this systematic review of 162 patients from two RCTs, pooled outcomes on the use of Omav for FA showed a time-dependent benefit relative to the placebo (PBO) group or baseline.

In these studies, Omav use was linked to significantly improved neurological functions, as assessed by improved mFARS scores in all four subsets, with maximal effects on upper limb subscores and upright stability [23] relative to PBO. However, values for individual components of mFARS were not provided in any of the included articles. Omav treatment, especially through EW 144, had a persistent benefit for FA patients compared to those who received Omav only after week 52. The mFARS values showed slow disease progression and improved neurological function after week 12 through EW 144 Omav treatment for more than 2.5 years [23, 24] in patients randomized to Omav through week 48. It could also be a preferential effect and may be a result of the greater maximum scores in these subsets [23, 26]. Although an increase in mFARS scores was reported in both RCTs, high standard deviations in each subset of patients are still a point of concern, which accounts for studies in large cohorts (Fig. 3).

The presence of pes cavus is generally associated with FA; however, the majority of patients in these studies did not have pes cavus (Table 5). GAA1 length is proportional to the severity of FA; however, in these studies, due to unclear reasons, patients with pes cavus had shorter GAA1 lengths. These studies particularly excluded patients with severe foot deformity [23]. This could suggest a longer duration for the treatment of FA with pes cavus by Omav and could be a contributing factor to the greater improvement in the mFARS score (Fig. 3) in patients without pes cavus than in those with pes cavus [23]. The anatomical location of action of Omav is doubtful, and thus, data from FA patients with pes cavus may not hold weightage with respect to clinical symptoms. In addition, patients with pes cavus were not balanced between the PBO and Omav groups, leading to probable artifactual connotations. Moreover, Omav demonstrated a greater response in those with longer GAA repeat lengths and in younger FA patients.

Overall, in a research analysis performed by the same group of researchers on the MOXIe study demonstrated a reduction of mFARS by 55% in the Omav group when matched controls of Friedreich Ataxia Clinical Outcome Measures Study (FACOMS) [27]. Considering FA a progressive disease, the indicated placebo effect was minimal after one year. Besides, the study suggested > 50% slow progression in Omav group through EW 144 at each year over 3 years when compared to the FACOMS [27].

Omav treatment was compatible across several clinical measures based on patients’ feelings and daily life functioning. The treatment efficacy was not significantly noticeable at 12 weeks; therefore, an extended duration of treatment with a larger cohort was used, as shown in week 48 and week 144. Nonetheless, secondary endpoints, including peak work during maximal exercise testing, frequency of falls, 9-HPT, T25-FW, CGIC, PGIC, and FA-ADL scores (using the FA-validated ADL questionnaire), did not significantly change from baseline, suggesting an extended treatment duration.

The safety profile was similar throughout the studies irrespective of the duration of the treatment. Omav was well tolerated by FA patients. Omav demonstrated Nrf2 activation by altering AST and GTT without affecting hepatic burden [10, 23]. This trend was similar to the response of healthy subjects on high-calorie and high-carbohydrate diets [10]. There were mainly mild adverse events in the initial treatment period, which could be due to adjustment to Omav. A few SAEs were also reported (Fig. 4), and a few patients discontinued treatment by week 48; however, adverse events were not reported as the reason for discontinuation of treatment by any subject after week 52 [10, 23, 24].

The studies were limited in terms of missing mFARS data during the COVID-19 pandemic, which impacted clinical visits, a small sample size, and the likelihood of unmeasured confounding effects beyond the EW 48. Nevertheless, missing visits are unlikely to bias the study. Although the effects of Omav were noticeable in patients aged younger than 18 years, a safety study was not performed on this age group of patients.

The present review included all the studies available for treating FA using Omav, although the studies were limited, and heterogeneity in the studies was negligible to mild. These data support the safety and clinical efficacy of Omav in FA patients. However, our study is limited due to the small number of original studies and clinical trials on the use of Omav for FA. Different levels of disease severity could lead to heterogeneity in outcomes; however, they have also not been addressed in studies. The duration of treatment and follow-up were variable among studies; however, continuation of the study may have limitations in terms of the data in the published literature. Severe adverse events, including COVID-19, may be related to SARS-CoV-2 and not primarily due to Omav treatment. Data published on pharmacokinetics and pharmacodynamics were not included in this study and provide a comprehensive overview of the safety and therapeutic efficacy of Omav in FA patients to the best of our knowledge. Therefore, considering these limitations, the results of this study should be interpreted with caution.

## Conclusion

This systematic review is an attempt to determine the safety and efficacy of Omav treatment in FA patients by assessing the quality of available evidence. Omav treatment does not result in mortality (0%); however, some SAEs were reported without casualty assessment. Adverse events were common in the PBO and Omav groups, with minimal SAEs. Furthermore, in all studies, the efficacy of Omav was assessed using the mFARS score. Although FNM was performed at week 12 as a primary outcome, the changes were not significant. The results of the Omav treatment indicated a persistent benefit to FA patients who received Omav for an extended period. The greater effect on longer GAA lengths than shorter repeats and the lower degree of improvement in pes cavus patients suggest that Omav aims for the most severe or progressive abnormalities in FA patients in a time-dependent manner.

Therefore, based on the available literature and the quality of the studies, further randomized controlled trials may include patients with progressive disease with the consideration of pes cavus, foot deformity. Considering the progressive nature of the disease, long-term follow-up beyond three years to constantly evaluate the effect of Omav on FA patients shall be considered. Direct neuromuscular assessments, such as peak workload, exercise testing, T25-FW, and 9-HPT, are needed to be assessed as primary outcomes in clinical trials. The neuronal substrates in pes cavus cases and the anatomical sites of action of Omav deserve further investigation. Clinical trials or interventional studies from other research or medical groups with all-inclusive data collection and reporting will be insightful and represent true assessment of the data.

## Supplementary Information

Additional file 1: is in .pdf format with the title “PICOS framework vis a vis use of omaveloxolone in Friedreich’s Ataxia”, which describes the population, intervention, comparator, outcome and study design in the records included in the manuscript.

Additional file 2: is in .pdf format with the title “Search Strategy” describing the strategy used to search the records in PubMed, Cochrane and Google Scholar.

## Data Availability

All data generated or analyzed during this study are included in this published article [and additional files].

## Abbreviations

9-HPT: Nine-hole peg test
ALT: Alanine transaminase
AST: Aspartate aminotransferase
BMP: B-type natriuretic peptide
CGIC: Clinician global impression of change
CHMP: Committee for Medicinal Products for Human Use
CTCRD: Central Technical Committee for Rare Diseases
EW: Extended week
FA: Friedreich’s ataxia
FA-ADL: Friedreich’s ataxia-activity of daily living scale
FACOMS: Friedreich’s Ataxia Clinical Outcome Measures Study
FNM: Functional neuromuscular assessment
HbA1c: Hemoglobin A1C test
ICMR: Indian Council of Medical Research
mFARS: Modified Friedreich’s Ataxia Rating Scale
NLM: National Library of Medicine
NPRD: National Policy for Rare Diseases
Nrf2: Nuclear factor erythroid 2-related factor 2
Omav: Omaveloxolone
PBO: Placebo
PGIC: Patient global impression of change
PRISMA: Preferred Reporting Items for Systematic Reviews and Meta-Analyses
PROSPERO: International Prospective Registry of Systematic Reviews
RCT: Randomized clinical trial
RoB2: Revised Cochrane risk-of-bias tool
Rpm: Rotation per minute
SAEs: Serious adverse events
T25-FW: Timed 25-foot walk test
WHO-ICTRP: World Health Organization’s International Clinical Trials Registry Platform
