# Supplementary material for: Safety and efficacy of omaveloxolone v/s placebo for the treatment of Friedreich's ataxia in patients aged more than 16 years: a systematic review
Source: Orphanet J Rare Dis. 2024 Dec 30;19:495. doi: 10.1186/s13023-024-03474-6 (PMC11684145; doi:10.1186/s13023-024-03474-6)
Supplement: Supplementary file 1 — Additional file 1: is in .pdf format with the title “PICOS framework vis a vis use of omaveloxolone in Friedreich’s Ataxia”, which describes the population, intervention, comparator, outcome and study design in the records included in the manuscript. [file 13023_2024_3474_MOESM1_ESM.pdf]

Additional file 1 PICOS framework vis a vis use of omaveloxolone in Friedreich's ataxia

**Name of the Component**	**Concepts**	**Keywords**
Population	Achondroplasia patients	Achondroplasia patients, rare disease, genetic diseases, rare disorder, autosomal-recessive
Intervention or Indicator (or cause, prognosis)	Omaveloxolone	Omaveloxolone
Comparison	Placebo	Not required
Outcome	Safety and efficacy	Not required
Study design	Interventional studies	Clinical trials, intervention, case studies
