# Supplementary material for: Safety and efficacy of omaveloxolone v/s placebo for the treatment of Friedreich's ataxia in patients aged more than 16 years: a systematic review
Source: Orphanet J Rare Dis. 2024 Dec 30;19:495. doi: 10.1186/s13023-024-03474-6 (PMC11684145; doi:10.1186/s13023-024-03474-6)
Supplement: Supplementary file 2 — Additional file 2: is in .pdf format with the title “Search Strategy” describing the strategy used to search the records in PubMed, Cochrane and Google Scholar. [file 13023_2024_3474_MOESM2_ESM.pdf]

Additional file 2 Search Strategy

**I. PubMed**		
**Syntax**	**Search terms**	**Result**
**#1**	((((("Friedreich's Ataxia patients") OR ("Friedreich's Ataxia")) OR ("Friedreich's Ataxia"[MeSH Major Topic])) OR ("Friedreich's Ataxia"[MeSH Subheading])) OR ("Friedreich's Ataxia"[MeSH Terms])) OR (Friedreich's Ataxia[MeSH Terms])	3,573
**#2**	(((((((genetic diseases) OR (genetic diseases[MeSH Terms])) OR (autosomal-recessive[MeSH Terms])) OR (autosomal-recessive)) OR (rare disorders)) OR (rare disorders[MeSH Terms])) OR ("rare diseases"[MeSH Terms])) OR (rare diseases)	1,357,367
**#3**	**#1 OR #2**	1,358,896
**#4**	(((Omaveloxolone) OR (Omaveloxolone[MeSH Terms])) OR (Omaveloxolone[MeSH Subheading])) OR (Omaveloxolone[MeSH Major Topic])	65
**#5**	**#3 AND #4**	**23**
**II. Cochrane**		
#1	Adults with filter = **word variations**	870948
#2	**Friedreich Ataxia** with filter = **word variations**	177
#3	Omaveloxolone with filter = **word variations**	14
#4	#2 AND #3	8
#5	#4 AND #1	**5**
**III. Google Scholar**		
#1	allintitle: Omaveloxolone OR Friedreich Ataxia	1480
#2	Omaveloxolone AND Friedreich Ataxia OR rare OR genetic disorders AND adults OR "young adults"	**167**
